# A draft genome for *Spatholobus suberectus*

**DOI:** 10.1038/s41597-019-0110-x

**Published:** 2019-07-04

**Authors:** Shuangshuang Qin, Lingqing Wu, Kunhua Wei, Ying Liang, Zhijun Song, Xiaolei Zhou, Shuo Wang, Mingjie Li, Qinghua Wu, Kaijian Zhang, Yuanyuan Hui, Shuying Wang, Jianhua Miao, Zhongyi Zhang

**Affiliations:** 1 College of Crop Science, Fujian Agriculture and Forestry University, Fuzhou, 350002 China; 2 Guangxi Key Laboratory of Medicinal Resources Protection and Genetic Improvement, Guangxi Botanical Garden of Medicinal Plants, Nanning, 530023 China; 3 Novogene Bioinformatics Institute, Beijing, 100083 China; 4 Key Laboratory of Genetics, Breeding and Comprehensive Utilization of Crops, Ministry of Education, Fujian Agriculture and Forestry University, Fuzhou, 350002 China

**Keywords:** Plant sciences, Genomics, Genome, DNA sequencing

## Abstract

*Spatholobus suberectus Dunn* (*S*. *suberectus*), which belongs to the Leguminosae, is an important medicinal plant in China. Owing to its long growth cycle and increased use in human medicine, wild resources of *S*. *suberectus* have decreased rapidly and may be on the verge of extinction. *De novo* assembly of the whole *S*. *suberectus* genome provides us a critical potential resource towards biosynthesis of the main bioactive components and seed development regulation mechanism of this plant. Utilizing several sequencing technologies such as Illumina HiSeq X Ten, single-molecule real-time sequencing, 10x Genomics, as well as new assembly techniques such as FALCON and chromatin interaction mapping (Hi-C), we assembled a chromosome-scale genome about 798 Mb in size. In total, 748 Mb (93.73%) of the contig sequences were anchored onto nine chromosomes with the longest scaffold being 103.57 Mb. Further annotation analyses predicted 31,634 protein-coding genes, of which 93.9% have been functionally annotated. All data generated in this study is available in public databases.

## Abstract (summary)

**Table:** 

Design Type(s)	sequence assembly objective • sequence annotation objective
Measurement Type(s)	whole genome sequencing assay
Technology Type(s)	DNA sequencing
Factor Type(s)	growth condition
Sample Characteristic(s)	Spatholobus suberectus • leaf

Machine-accessible metadata file describing the reported data (ISA-Tab format)

## Background & Summary

**Fig. 1:**
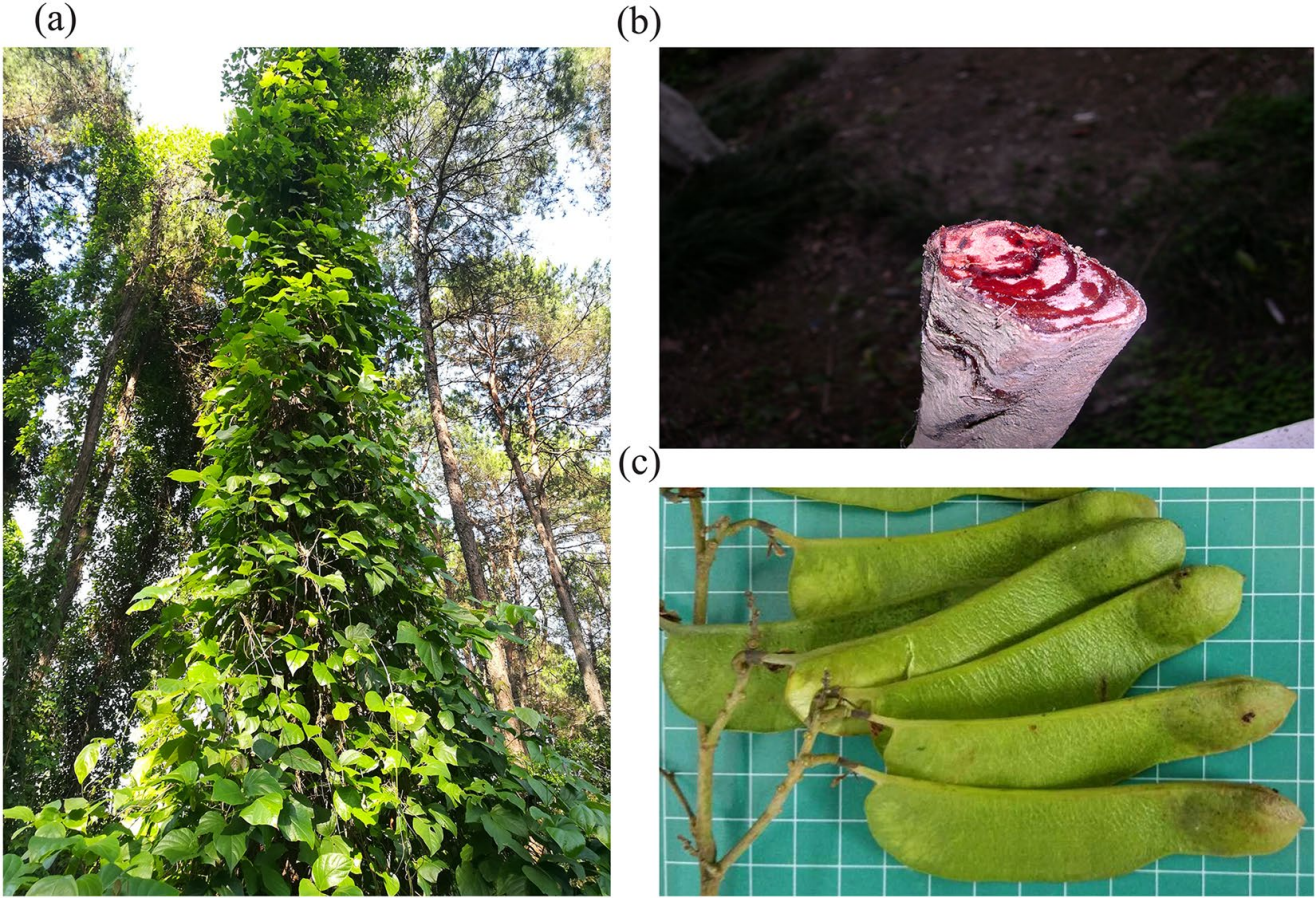
Morphological character of *S*. *suberectus*. (**a**) A picture of *S*. *suberectus* plant. (**b**) The vine stem of *S*. *suberectus* is called “chicken blood vines”. (**c**) The pod of *S*. *suberectus* has only one seed.

*Spatholobus suberectus* Dunn is widely used as a food supplement in tea, wine, and soup as well as being one of the most important Chinese medicinal plants (Fig. 1a) for treatment of various diseases such as blood stasis syndrome, abnormal menstruation, and rheumatism^1^. It is mainly distributed in Fujian, Guangdong, Yunnan Province, and the Guangxi Zhuang Autonomous Region of China^2^. The vine stem of *S*. *suberectus*, called “chicken blood vines” in China due to an outflow of red juice outflow when the vine stem is injured (Fig. 1b), is the critical medicinal component^3^. Pharmacological and clinical studies have demonstrated that *S*. *suberectus* exhibits various functions against oxidation^4^, viruses^5^, bacteria^6^, cancer^7^, and platelets^8^. The crud drug of *S*. *suberectus* is therefore used in many patented Chinese medicines, and the market demand for the wild resource is increasing rapidly. But unlike other Leguminosae plants, the seed setting rate of *S*. *suberectus* is low (Fig. 1c), and most of the fruit falls off before seed maturation, which results in a low natural reproductive capacity. The growth cycle of *S*. *suberectus* is very long and the crud drug must grow for more than seven years before it can be used as medicine. These factors have combined to decrease the wild resources of *S*. *suberectus* in China to the verge of extinction.

To investigate biosynthesis of the main bioactive components and seed development mechanism needed for future *S*. *suberectus* production we generated a high-quality draft version of the *S*. *suberectus* genome. Whole-genome sequencing of several species in Leguminosae plants have been performed, for instance, *Lotus japonicus*^9^, *Glycine max*^10^, *Medicago truncatula*^11^, *Glycyrrhiza uralensis*^12^, *Cicer arietinum*^13^, and *Cajanus cajan*^14^, however, there are few reports of Subtribe Erythrininae Benth, containing nine genera of Leguminosae^2^. As one of the members of this subtribe, genomic information of *S*. *suberectus* can fill this gap.

The genome size of *S*. *suberectus*, a diploid (2n = 18) species, was estimated to be 793 Mb using 17-mer frequency distribution analysis with SOAPdenovo. In this study, we combined sequences generated on the Illumina, PacBio, and 10X Genomics GemCode platform as well as the new assembly technique FALCON to generate the first draft genome assembly of *S*. *suberectus*. The assembled genome is about 798 Mb with scaffold and contig N50 sizes of 6.9 Mb and 2.1 Mb, respectively. The *S*. *suberectus* assembly was further refined using 233.19 Gb Hi-C data: 748 Mb (93.73%) of the contig sequences were anchored onto nine chromosomes, the scaffold N50 was improved to be 86.99 Mb, and the longest scaffold was 103.57 Mb.

Almost half of the *S*. *suberectus* genome (47.82%) was occupied by repetitive elements, the largest amount of which was long terminal repeat retrotransposons (17.32%). Combined with homology-based predictions, *de novo* predictions and transcriptome-based predictions, 31,634 protein-coding genes with an average transcript size of 1,097.55 bp were predicted in the genome. In total, 93.9% (29,688) of protein-coding genes were successfully functionally annotated.

## Methods

### Plant materials and DNA extraction

*S*. *suberectus* samples from Nanning, Guangxi Zhuang Autonomous Region, China (22°51′28″N, 108°22′2″E) were selected for genome sequencing. The samples were kept at the Guangxi Botanical Garden of Medicinal Plants for breeding and research purposes. Total genomic DNA was isolated from fresh young leaves of 8-year-old *S*. *suberectus* using the Plant DNA Kit (TIANGEN) according to the manufacturer’s instructions.

### Library construction and sequencing

The DNA was sheared by a Covaris® M220 focused-ultrasonicator^TM^ (Covaris, Woburn, Massachusetts, USA). The sheared DNA, with fragment sizes of 250 bp and 450 bp, was processed using the TrueSeq DNA PCR-Free LT Library Kit protocol. PCR products were purified (AMPure XP system) and library quality was assessed on an Agilent Bioanalyzer 2100 system. These PCR-Free libraries were sequenced with a HiSeq X Ten instrument as 150 bp paired-end reads. In total, 77.73 Gb of raw sequence data were generated (Table 1).

**Table 1:** The sizes of sequencing data using various sequencing platforms.

Pair-end libraries	Platform	Insert size	Total Data(G)	Read length (bp)	Sequence Coverage(X)
Illumina	Illumina HiSeq	250 bp	41.89	150	52.82
		450 bp	35.84	150	45.20
Pacbio reads	Pacbio Sequel	20 kb	63.27	—	79.79
10×	Illumina HiSeq	20 kb	123.09	150	155.22
Hi-C	Illumina HiSeq	350 bp	233.19	150	293.92

Sheared DNA (40 μg) was purified and concentrated with AMPure PB beads (PacBio) and further used for SMRTbell preparation according to the manufacturer’s protocol (Pacific Biosciences; 20-kb template preparation using BluePippin (Sage Science) size selection system with a 15-kb cut-off). The libraries were then sequenced with a PacBio sequel instrument (Pacific Biosciences, Menlo 31 Park, CA, USA). A total of 11 SMRT Cells were used to yield 79.79-fold genome coverage of sequence data (Table 1), consisting of 63.27 Gb sequence data with an N50 read length of 14,288 bp (Table 2).

**Table 2:** Statistics of characteristics of Pacbio long-read.

Read_type	Read_base	Read_Number	Read_length (max)	Read_length (mean)	Read_length (N50)
Subreads	63,270,110,556	6,710,707	122,873	9,428	14,288

The linked read sequencing libraries were constructed on a 10X Genomics GemCode platform^15^. Sample indexing and partition barcoded libraries were prepared using the Chromium Genome Reagent Kit (10x Genomics) according to the manufacturer’s instructions. The barcode sequencing library was first quantified by Qubit2.0, insert size was checked using an Agilent2100, and finally quantified by qPCR. The 123.09 Gb library was sequenced with 150 bp paired-end reads on an Illumina HiSeq X Ten platform (Table 1).

For the Hi-C library, chromatin was fixed in place with formaldehyde in the nucleus. Fixed chromatin was digested with DpnII restriction endonuclease, 5′ overhangs were filled in with biotinylated nucleotides, and free blunt ends were ligated. After ligation, cross-links were reversed, and the DNA was purified from protein. Purified DNA was treated to remove biotin that was not internal to the ligated fragments. The DNA was then sheared to a mean fragment size of 350 bp, and sequencing libraries were generated using NEBNext Ultra enzymes and Illumina-compatible adaptors. Biotin-containing fragments were isolated using streptavidin beads before PCR enrichment of each library. The libraries were sequenced on an Illumina HiSeq platform to produce 233.19 Gb Hi-C sequence data (Table 1). The quality of Hi-C sequencing was evaluated using HiCUP^16^. The effect rate (%) = Unique di-tigs/Total Reads Processed = 4,356,614/10,000,000 = 43.57% (Table 3). Typically, 35.91% of the alignable read pairs represent interchromosomal interactions. Eleven percent represents intrachromosomal interactions between fragments less than 10 kb apart and 53.09% are intrachromosomal read pairs that are more than 10 kb apart (Table 3).

**Table 3:** Statistics of Hi-C sequencing and mapping.

Statistics of mapping		
	Read1	Read2
Total Reads	10,000,000	10,000,000
Unique Alignments	7,869,514	7,702,126
Multiple Alignments	859,867	832,203
Failed To Align	866,895	1,073,869
Unique Mapped Paired-end Reads	6,056,459	6,056,459
**Statistics of valid reads**		
Unique Mapped Paired-end Reads	6,056,459	
Invalid Paired-end Pairs	1,699,845	
Valid Paired-end Reads	4,356,614	
Valid Rate (%)	43.56	
Cis-close (<10 Kbp)	478,994	
Cis-far (>10 Kbp)	2,313,017	
Trans	1,564,603	

Cis-close (<10 Kbp): interactions between intrachromosomal read pairs less than 10 kb apart.

Cis-far (>10 Kbp): interactions between intrachromosomal read pairs more than 10 kb apart.

Trans: the alignable read pairs represent interchromosomal interactions.

### Estimation of the *S*. *suberectus* genome size

**Fig. 2:**
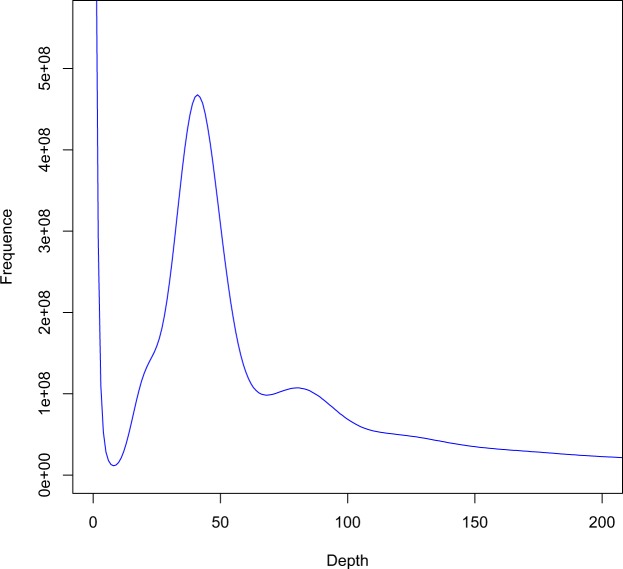
Estimation of *S*. *suberectus* genome size by K-mer analysis.

Quality-filtered reads from the Illumina platform were subjected to 17-mer frequency distribution analysis with SOAPdenovo^17^. K-mer 17 was selected to estimate the genome size and heterozygosity of *S*. *suberectus* (Fig. 2). We plotted the distribution of k-mer depth against frequency with a main peak occurring at the depth of 40 (Fig. 2). Based on the total number of k-mers (32,476,446,092), the *S*. *suberectus* genome size was calculated to be approximately 793.39 Mb, using the following formula: genome size = k-mer_Number/Peak_Depth and Revised Gsize = Genome size × (1-Error Rate). The heterozygosity of the *S*. *suberectus* genome is 0.74%.

### Genome assembly

*De novo* assembly of the 63.27 Gb PacBio single-molecule long reads from SMRT Sequencing was performed using FALCON (https://github.com/PacificBiosciences/FALCON/)^18^. In order to get enough corrected reads, the longest 60 subreads were first selected as seed reads to do error correction. Then error-corrected reads were aligned to each other and assembled into genomic contigs using FALCON with parameters length_cutoff_pr = 5000, max_diff = 120, max_cov = 130. The draft assembly was polished using the quiver algorithm. Pilon was used to perform error correction of p-contigs with 98.02X coverage of short paired-end reads generated from Illumina HiSeq Platforms^19^. The assembly consisted of 1,954 contigs, with a contig N50 length of 2.06 Mb (total length = 794 Mb) (Table 4).

**Table 4:** Summary of *S*. *suberectus* genome assembly using PacBio long reads.

Sample ID	Length	Number
	Contig (bp)	Contig
Total	794,088,373	1,954
Max	8,229,915	—
Number >=2000	—	1,928
N50	2,057,658	114
N60	1,446,732	161
N70	1,036,389	226
N80	673,988	322

We used BWA-MEM^20^ to align the 10X Genomics data to the assembly using default settings. Scaffolding was performed by fragScaff (*in vitro*, long-range sequence information for *de novo* genome assembly via transposase contiguity) with the barcoded sequencing reads.

The assembly consisted of 1,146 scaffolds, with the scaffold N50 length improving to 6.9 Mb (total length = 798 Mb) and contig N50 of 2.1 Mb (Table 5). The genome assembly size is similar to the estimated genome size by k-mer analysis.

**Table 5:** Summary of *S*. *suberectus* genome assembly using PacBio long reads and 10X genomics data.

Sample ID	Length		Number	
	Contig (bp)	Scaffold(bp)	Contig	Scaffold
Total	794,088,373	798,435,360	1,954	1,146
Max	8,229,915	27,701,983	—	—
Number >=2000	—	—	1,928	1,120
N50	2,057,658	6,903,381	114	34
N60	1,446,732	5,179,305	161	47
N70	1,036,389	3,931,704	226	64
N80	673,988	2,630,391	322	89

The input *de novo* assembly, shotgun reads, and Dovetail Hi-C library reads were used as input data for HiRise, a software pipeline designed specifically for using proximity ligation data to scaffold genome assemblies^21^. Shotgun and Dovetail Hi-C library sequences were aligned to the draft input assembly using a modified SNAP read mapper (http://snap.cs.berkeley.edu). The separations of Dovetail Hi-C read pairs mapped within draft scaffolds were analyzed by HiRise to produce a likelihood model for genomic distance between read pairs, and the model was used to identify and break putative misjoins, score prospective joins, and make joins above a threshold. After scaffolding, shotgun sequences were used to close gaps between contigs.

**Fig. 3:**
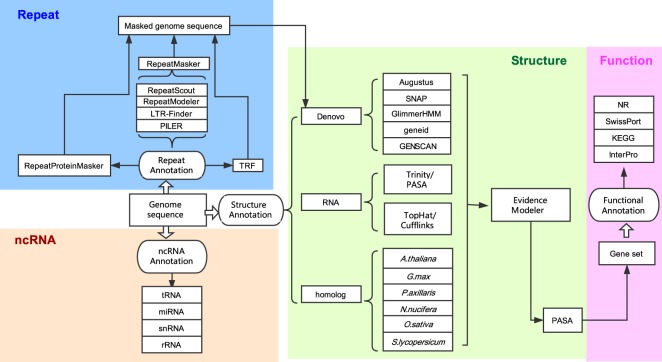
Diagrammatic sketch of the annotation pipeline.

The *S*. *suberectus* assembly was further refined using 233.19 Gb Hi-C data (Table 1): 748 Mb (93.73%) of the contig sequences were anchored onto nine chromosomes (Fig. 3). The scaffold N50 was finally improved to be 86.99 Mb and the longest scaffold was 103.57 Mb.

### Indentification of repetitive elements in *S*. *suberectus*

Tandem Repeat Finder^22^ was employed to identify tandem repeats in the *S*. *suberectus* genome. RepeatMasker (http://www.repeatmasker.org) and RepeatProteinMasker^23^ were used against Repbase^24^ to identify known transposable element repeats. In addition, RepeatModeler (http://www.repeatmasker.org/RepeatModeler.html), RepeatScout (http://www.repeatmasker.org/)^25^, PILER (http://www.drive5.com/piler/)^26^, and LTR_Finder (http://tlife.fudan.edu.cn/ltr_finder)^27^ were utilized to identify *de novo* evolved repeats (Fig. 3).

**Fig. 4:**
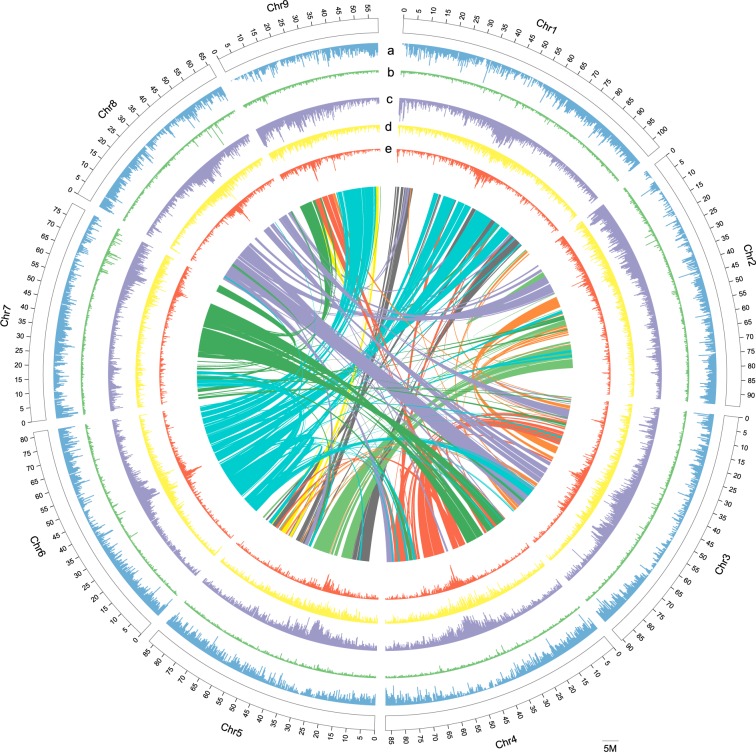
Circos Plot Showing the Genomic Features of *S*. *suberectus*. Concentric circles, from outermost to innermost, show (**a**) gene density (blue), (**b**) tandem repeats density (green), (**c**) transposon element density (purple), (**d**) LTR-Copia density (yellow), (**e**) LTR-Gypsy density (red) and intra-genome collinear blocks connected by curved lines. All distributions are drawn in a window size of 300 kb, chromosomes_ scale = 5,000,000 bp.

The combined results show that almost half of the *S*. *suberectus* genome (47.82%) was occupied by repetitive elements (Fig. 4b–e). Among these, long terminal repeat (LTR) retrotransponsons represent the largest amount of repetitive elements, reaching 17.32% of the genome, fewer than soybean (42%)^10^ and chickpea (46%)^28^, but are similar to *Lotus japonicus* (18%)^9^. LTR/Copia repeats were the most abundant, making up 10.06% of the genome (Fig. 4d), followed by LTR/Gypsy elements (6.61%; Fig. 4e).

### Gene annotation

Genes in the *S*. *suberectus* genome were annotated using multiple methods, including homology-based predictions, *de novo* predictions and transcriptome-based predictions (Fig. 3). For *de novo* predictions, Augustus^29^, GENSCAN^30^, GlimmerHMM^31^, geneid^32^ and SNAP^33^ analysis were performed on the repeat-masked genome, with parameters trained from *Arabidopsis thaliana*. Predicted protein sequences from *Nelumbo nucifera* (ftp://ftp.ncbi.nlm.nih.gov/genomes/refseq/plant/Nelumbo_nucifera/latest_assembly_versions/GCF_000365185.1_Chinese_Lotus_1.1 version 1.1), *Arabidopsis thaliana* (ftp://ftp.ensemblgenomes.org/pub/plants/release-32/gff3/arabidopsis_thaliana/, version 10.32), *Glycine max* (ftp://ftp.ensemblgenomes.org/pub/plants/release-32/fasta/glycine_max/dna/, version 1.0), *Petunia axillaris* (ftp://ftp.solgenomics.net/genomes/Petunia_axillaris/, version 1.6.2), *Solanum lycopersicum* (ftp://ftp.ensemblgenomes.org/pub/plants/release-32/fasta/solanum_lycopersicum/, release-32), and *Oryza sativa* (ftp://ftp.ensemblgenomes.org/pub/plants/release-32/fasta/oryza_sativa/, version 1.0) were used for homology-based predictions. First, query sequences were subjected to tblastn analysis with an Expect (E)-value cutoff of 1e-5. BLAST hits corresponding to reference proteins were concatenated by Solar software, and low-quality records were removed. The genomic sequence of each reference protein was extended upstream and downstream by 2,000 bp to represent a protein-coding region. Gene structures contained in each protein region were predicted using GeneWise software^34^. For transcriptome-based predictions, RNA from five organs (root, petiole, leaves, flowers, and stems) was isolated and RNA-seq data were used for gene annotation, processed by TopHat and Cufflinks^35^. RNA-seq data were also assembled by Trinity^36^. PASA^37^ software (http://pasapipeline.github.io/) was then used to generate a full transcriptome-based genome annotation. The homology, *de novo*, and transcriptomic gene sets were merged to form a comprehensive and non-redundant reference gene set using EVidenceModeler^38^ software. Next, PASA^37^ was used to generate UTRs as suggested by the RNA-seq data.

Our analysis indicates that 31,634 protein-coding genes with an average transcript size of 1,097.55 bp were predicted in the genome (Fig. 4a).

Functional annotation of the protein-coding genes was carried out using blastp (E-value cut-off 1e**-**05) against SwissProt^39^ and NR databases. Protein domains were annotated by searching against InterPro^40^ and Pfam database^41^, using InterProScan and HMMER (http://hmmer.janelia.org), respectively. The GO terms for genes were obtained from the corresponding InterPro or Pfam entry. The pathways in which the genes might be involved were assigned by BLAST against the KEGG database^42^ with the E-value cut-off of 1e-05.

Overall, 79% (24,976), 70.8% (22,394), and 82.5% (26,082) of genes showed enrichment in InterPro, KEGG, and GO respectively. In total, 93.9% (29,688) of protein-coding genes were successfully annotated for conserved functional motifs or functional terms.

### Non-coding RNA annotation

Annotation of tRNA was performed using tRNAscan-SE^43^ software with default parameters. rRNA annotation was based on homology with rRNAs from several diverse higher plant species (not shown), using blastn with ‘E-value = 1e-5’. miRNA and snRNA genes were predicted by INFERNAL software^44^ using the Rfam database^45^.

The final results included 820 miRNA, 672 tRNA, 261 rRNA, and 550 snRNA with average lengths of 117.33, 75.32, 305.41 and 115.50 bp respectively.

## Data Records

This Whole Genome Shotgun project has been deposited at DDBJ/ENA/GenBank under the accession QUWT00000000^46^. The version described in this paper is version QUWT01000000. Raw read files are available at NCBI Sequence Read Archive^47^. All the annotation tables containing results of an analysis of the draft genome are available at figshare^48^.

## Technical Validation

### Evaluation of the completeness of the *S*. *suberectus* genome assembly

To estimate the quality of genome assembly, short reads were mapped back to the consensus genome using BWA^49^ and an overall 97.29% mapping rate was found, suggesting that our assembly results contained comprehensive genomic information. Gene region completeness was evaluated by RNA-Seq data (Table S1): of the 53,538 transcripts assembled by Trinity^36^, 99.62% could be mapped to our genome assembly, and 95.94% were considered as complete (more than 90% of the transcript could be aligned to one continuous scaffold).

The completeness of gene regions was further assessed using CEGMA (conserved core eukaryotic gene mapping approach)^50^: 240 of 248 (96.77%) conserved core eukaryotic genes from CEGMA were captured in our assembly, and 206 (83.06%) of these were complete (Table S2). Furthermore, we performed BUSCO (Benchmarking Universal Single-Copy)^51^ analysis based on a benchmark of 956 conserved plant genes, of which 96% had complete gene coverage (including 18% duplicated ones), 1% were fragmented and only 2.6% were missing (Table S3). These data largely support a high quality *S*. *suberectus* genome assembly, which can be used for further investigation.

## Supplementary Information

### ISA-Tab metadata file

Download metadata file

### Supplementary information

Supplementary Tables

## Data Availability

The execution of this work involved many software tools, whose versions, settings and parameters are described below. **(1) SOAPdenovo:** version 3.0, default parameters; **(2) FALCON:** version 3.1, length_cutoff_pr = 5000, max_diff = 120, max_cov = 130; **(3) HiCUP:** version 0.5.10, **(4) HiRise:** Dovetail Genomics LLC, Santa Cruz, CA, USA; **(5) BWA:** version 0.7.8, default parameters; **(6) Tandem Repeat Finder:** version 409, default parameters; **(7) RepeatMasker:** version 4.0.5, default parameters; **(8) Repbase:** version 15.02; **(9) RepeatModeler:** version 1.0.11, default parameters; **(10) RepeatScout:** version 1.0.5, default parameters; **(11) PILER:** version 1.06, default parameters; **(12) LTR_FINDER:** version 1.0.7, default parameters; **(13) Augustus:** version 3.0.2, default parameters; **(14) GENSCAN:** version 1.0, default parameters; **(15) geneid:** version 1.4, default parameters; **(16) GlimmerHMM:** version 3.0.2, default parameters; **(17) SNAP:** version 11-29-2013; **(18) BLAST:** version 2.2.26, default parameters; **(19) GeneWise:** version 2.2.0, default parameters; **(20) TopHat:** version 2.0.8, default parameters; **(21) Cufflinks:** version 2.1.1, default parameters; **(22) Trinity:** version 2.4.0, default parameters; **(23) PASA:** version 2.3.3, default parameters; **(24) EVidenceModeler:** version 1.1.1, default parameters; **(25) InterPro:** version 5.16, default parameters; **(26) Pfam database:** version 03-30-2016; **(27) InterProScan:** version 4.8, default parameters; **(28) NR database:** version 08-10-2015; **(29) KEGG database:** version 08-31-2015; **(30) SwissProt database:** version 05-24-2016; **(31) HMMER:** version 3.1b1, default parameters; **(32) tRNAscan-SE:** version 1.3.1, default parameters; **(33) BUSCO**: version 3.0.2, Embryophyta Version odb9; **(34) CEGMA:** version 2.5.
