# Supplementary material for: A draft genome for Spatholobus suberectus
Source: Sci Data. 2019 Jul 4;6:113. doi: 10.1038/s41597-019-0110-x (PMC6609623; doi:10.1038/s41597-019-0110-x)
Supplement: Supplementary file 2 — Supplementary Tables [file 41597_2019_110_MOESM2_ESM.docx]

| **Dataset** | **Number** | **Total length (bp)** | **Sequences Covered by assembly** | **with >90% sequence in one scaffold** | | **with >50% sequence in one scaffold** | |
| --- | --- | --- | --- | --- | --- | --- | --- |
|  |  |  |  | **Number** | **Percent (%)** | **Number** | **Percent (%)** |
| >200bp | 53,538 | 32,278,647 | 99.621 | 51,362 | 95.936 | 53,047 | 99.083 |
| >500bp | 18,314 | 21,491,563 | 99.82 | 17,605 | 96.129 | 18,187 | 99.307 |
| >1 Kb | 7,893 | 14,316,279 | 99.911 | 7,617 | 96.503 | 7,865 | 99.645 |
| >2 Kb | 2,268 | 6,456,994 | 99.912 | 2,178 | 96.032 | 2,262 | 99.735 |

**Table S1. Assessment of gene coverage rate using EST data.** ESTs assembled by transcriptome data, sequenced from the *S. suberectus*, were aligned to the scaffolds, and 95.94% ESTs were over 90% covered in one scaffold.

| **Species** | **Complete** | | **Complete + partial** | |
| --- | --- | --- | --- | --- |
|  | **Prots** | **%completeness** | **Prots** | **%completeness** |
| *S. suberectus* | 206 | 83.06 | 240 | 96.77 |

**Table S2. CEGMA assessment of *S. suberectus* genome.** CEGMA defined the number of 248 ultra-conserved CEGs that occur in a wide range of eukaryotes. A protein is classified as complete if the alignment of the predicted protein to the HMM profile represents at least 70% of the original KOG domain, otherwise it is classified as partial.

| **Species** | **BUSCO notation assessment results** |
| --- | --- |
| *S. suberectus* | C:96%[D:18%], F:1.0%, M:2.6%, n:956 |

**Table S3. BUSCO notation assessment of *S. suberectus* genome.** BUSCO (Benchmarking Universal Single-Copy Orthologs: <http://busco.ezlab.org>) C: Complete Single-Copy BUSCOs; D: Complete Duplicated BUSCOs; F: Fragmented BUSCOs; M: Missing BUSCOs; n: Total BUSCO groups searched.
